# Area-Level Variation in Adolescent Smoking

**Published:** 2009-03-15

**Authors:** Debra H. Bernat, DeAnn Lazovich, Jean L. Forster, J. Michael Oakes, Vincent Chen

**Affiliations:** School of Nursing, University of Minnesota; University of Minnesota, Minneapolis, Minnesota; University of Minnesota, Minneapolis, Minnesota; University of Minnesota, Minneapolis, Minnesota; University of Texas, Houston, Texas

## Abstract

**Introduction:**

The purpose of this study is to 1) examine the variability in the prevalence of adolescent smoking in 60 geographic areas of Minnesota and 2) assess how variability in area-level smoking prevalence is associated with area-level sociodemographic characteristics.

**Methods:**

Smoking data were collected from 3,636 adolescents residing in 60 areas of the state of Minnesota. Area-level characteristics were obtained from the 2000 US Census. Coefficient of variation was calculated to assess variability in smoking prevalence across areas, and mean smoking prevalence was compared above and below the median for each area-level characteristic.

**Results:**

Substantial variation was found in adolescent smoking prevalence rates. Across the 60 areas, the percentage of adolescents that ever smoked varied from 13% to 53%, and the percentage of adolescents that smoked in the past 30 days ranged from 3% to 19%. Mean lifetime smoking prevalence was higher in areas with a higher percentage of residents with less than a high school education, a lower percentage of residents living in an urban area, lower median housing value and a lower median household income, a higher percentage of residents aged 16 years or older who were unemployed, and a higher percentage of residents with an income-to-poverty ratio less than 1.5. Similar results were found for past 30-day smoking prevalence among girls; however, no area-level characteristics were significantly associated with past 30-day smoking prevalence among boys.

**Conclusion:**

Results suggest that area-level characteristics may play an important role in adolescent smoking, particularly for girls.

## Introduction

Youth tobacco use remains a challenging public health problem. Despite warnings about the long-term health consequences of tobacco use, many young people in the United States continue to smoke. Nationally, nearly one-half (47%) of all 12th-grade students report having smoked cigarettes in their lifetime (1). Additionally, 9% of eighth-grade students, 15% of 10th-grade students, and 22% of 12th-grade students report smoking cigarettes in the past 30 days.

Researchers are becoming increasingly aware of the complexity of smoking behavior, and determinants exist at both the macro and micro levels (2). Because of the substantial variation in the prevalence of tobacco use across groups and geographies, factors beyond the individual are now believed to play an important role in tobacco use. State-level surveillance data show that smoking prevalence among ninth through 12th graders ranges from 7% in Utah to 36% in Louisiana (3). Although few studies have examined small geographic areas, several studies suggest that smoking prevalence varies within states. One study showed that adult smoking rates ranged from 18% to 39% in 6 communities in Chicago (4). Another study found that 44% of Harlem residents aged 18 to 65 were current smokers, although only 25% of adults in the state of New York in this same age range were current smokers (5). Geographic variations in health outcomes are likely due to various factors, including area-level social norms, opportunity structures, environmental attributes, and simple demographic differences (6).

Understanding how both macro- and micro-level processes affect health behaviors is a challenge for social epidemiologists (6,7). Many studies have used multilevel regression models to disentangle the effects of macro- and micro-level factors on health behavior. However, serious methodological concerns about our ability to do this have been raised (6,8,9). In fact, Oakes (6) goes so far as to say it is practically impossible to disentangle such effects with observational data sets. Such concerns suggest that a better research strategy may be to focus on one level of analysis — either the individual or the group — but not both simultaneously (10). Straightforward ecological designs may be the best way forward (11-13).

This study used data from the Minnesota Adolescent Community Cohort (MACC)This study used data from the Minnesota Adolescent Community Cohort (MACC) Study to assess geographic variation in youth smoking rates and macro-level factors associated with variation in smoking prevalence rates. The goals of this study are to 1) examine variability in youth smoking rates for 60 randomly selected areas across Minnesota and 2) assess how variability in area-level prevalence of smoking is associated with area-level sociodemographic characteristics. Determining areas that have increased risk of youth smoking could provide useful information for organizations that implement tobacco use prevention programs (14).

## Methods

### Design

The data from the present study are from the MACC Study, which began in 2000. Before recruiting participants, the state was divided into area-level units, referred to as geopolitical units (GPUs)The data from the present study are from the MACC Study, which began in 2000. Before recruiting participants, the state was divided into area-level units, referred to as geopolitical units (GPUs), on the basis of geographical and political boundaries thought to reflect local tobacco control environments. We constructed these units on the basis of the following criteria: 1) the boundaries of the GPUs fell within existing geographic and/or political limits, 2) the boundaries of the GPUs reflected patterns of local tobacco program activities, and 3) a sufficient number of teenagers resided in each GPU to meet the sample size requirements. Using these criteria, the state was divided into 129 GPUs. Next, a stratified random sample of 60 GPUs was selected, on the basis of the region of the state in which the GPU was located and distribution of race/ethnicity by GPU. Of the 60 GPUs, 24 were defined by county boundaries, 28 were defined by school districts, and 8 were subunits of cities (including local planning districts). Among the 60 GPUs, 28 were rural (47%), 21 were suburban (35%), 3 were cities, not within the metropolitan area (5%), and 8 were urban (13%).

Once the GPUs were defined and selected, an equal number of youth for each year of age between 12 and 16 were recruited to participate from each GPU. A combination of probability and quota sampling methods was used to ensure equal age distribution. Participants were recruited by telephone by Clearwater Research, Inc, using a modified random-digit–dial method. A total of 200,849 households were called to identify those with at least 1 teenager between the ages of 12 and 16 years within target GPUs. Among 6,213 eligible households, respondents were selected at random from age quota cells that were still open for each GPU. With parental consent, a total of 3,636 (58.5%) adolescents completed a 10- to 20-minute interview between October 2000 and March 2001. Study participants received $10 for participating. Approximately an equal number of boys (n = 1,789; 49%) and girls (n = 1,847; 51%) were enrolled in the study. The University of Minnesota institutional review board approved this study, and all participants provided informed consent to participate.

### Measures

To describe the sociodemographic characteristics of the GPUs, we used Summary File 3 (15) from the US Census Bureau, which provides detailed information for a sample of the population at the census block group level. We selected the following sociodemographic characteristics thought to be related to smoking prevalence: percentage of population that was white, percentage with less than a high school education, percentage that was married, percentage living in an urban area, percentage aged 18 years or older that was English speaking, median housing value, percentage living in renter-occupied housing, median household income, percentage (aged 16 years and older) unemployed, and percentage living below the federal poverty level (income-to-poverty ratio <1.5). Since GPU boundaries could divide census block groups but contain intact census blocks, we first determined the proportion of the total population for each block group that was contained within the GPU by aggregating census blocks. We then used this value to adjust the numerator and denominator of those census characteristics expressed as proportions (eg, percentage of the population that was white) for each block group within the GPU to obtain the correct GPU-level proportion for a given census characteristic. For census characteristics expressed as a median (income, housing value), we took the average of the medians across all block groups within the GPU.

Two measures of tobacco use were derived from the youth telephone surveys. Lifetime smoking was defined as ever having smoked a cigarette, including taking a few puffs. Past 30-day smoking was defined as having smoked on 1 or more days during the past 30 days. GPU-level tobacco use prevalence was computed by calculating the percentage of youth in each GPU that reported lifetime and past 30-day smoking.

### Data analysis

All analyses were conducted at the GPU level (n = 60). First, we calculated the coefficient of variation (CV) to assess the variability in GPU sociodemographic characteristics and prevalence of smoking (lifetime and past 30-day) across the 60 GPUs. The CV was calculated as the standard deviation divided by the mean, expressed as a percentage; a high CV represents greater variability across GPUs. Next, using a median split specific to each sociodemographic characteristic, we conducted *t* tests to assess differences in the mean prevalence of lifetime and mean past 30-day smoking for GPUs above and below the median for each GPU characteristic examined. Because previous studies have shown that different factors predict smoking for men and women at the individual level (16-18), we tested whether results for mean smoking prevalence (lifetime or past 30-day) should be stratified by sex. Finally, we used multiple linear regression to assess the variability in GPU-level lifetime and past 30-day smoking that was explained by GPU sociodemographic characteristics. All GPU sociodemographic characteristics found to be statistically significantly related to GPU-level smoking at the bivariate level (*P* < .05) were included in the regression models.

## Results

### Coefficient of variation

The CV for the GPU sociodemographic characteristics ranged from 5.7% (percentage aged ≥18 y that was English speaking) to 55.4% (percentage with an income-to-poverty ratio <1.5) (Table 1). The coefficient of variation was 30% or above for most (7 of 10) of the GPU sociodemographic characteristics. Less variation was found for the percentage that was white, married, and English speaking (of those ≥18 years).

The percentage of adolescents that ever smoked varied from 13.3% to 53.3% across the 60 GPUs, with a mean of 33.1% (CV = 26%). The Figure shows the smoking prevalence rates in Minnesota by GPU. Mean prevalence of lifetime smoking was similar for boys (33.6%) and girls (32.5%) (*P* = .67) (data not shown). The prevalence of past 30-day smoking ranged from 3.2% to 18.6% across the 60 GPUs, with a mean of 9.5% (CV = 41.1%) (Table 1). The mean prevalence of past 30-day smoking was higher for girls (10.4%) than boys (8.6%) (*P* = .04) across the GPUs (data not shown).

**Figure 1:**
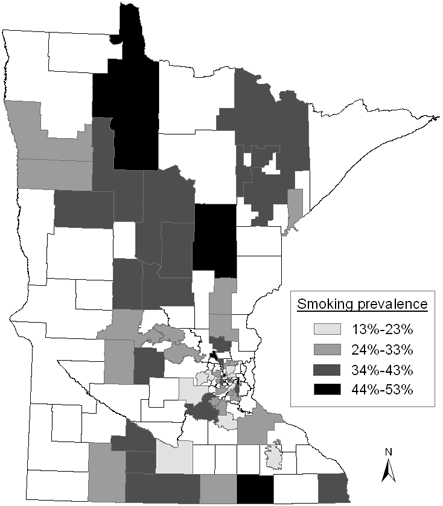
Lifetime smoking prevalence by geopolitical units (GPUs), Minnesota State, Minnesota Adolescent Community Cohort (MACC) Study, October 2000-March 2001. (Nonshaded areas are GPUs not selected for the MACC Study.)

**Table:** 

### Bivariate results

Mean lifetime smoking prevalence above and below the median for each GPU characteristic was similar for boys and girls, so bivariate results are reported for the entire sample (Table 2). Mean lifetime smoking prevalence was significantly higher in GPUs with a higher percentage of residents with less than a high school education, a lower percentage of residents living in an urban area, lower median housing value and a lower median household income, a higher percentage of residents aged 16 years or older who are unemployed, and a higher percentage of residents with an income-to-poverty ratio less than 1.5. The mean prevalence of lifetime smoking in GPUs with a higher percentage of English-speaking residents aged 18 years or older approached significance.

Patterns observed for past 30-day smoking differed for boys and girls, so results are reported separately. Among girls, we found differences in the mean prevalence of past 30-day smoking for above compared with below the median for all 10 GPU sociodemographic characteristics; for 6 of the 10, these differences were significant. Areas with a higher smoking prevalence among girls were GPUs with a higher percentage of residents with less than a high school degree, a lower percentage of residents living in an urban area, a lower median housing value, a lower median household income, a higher percentage of residents aged 16 years or older who were unemployed, and more residents with an income-to-poverty ratio less than 1.5. Among boys, none of the comparisons in mean smoking prevalence above and below the median value for any GPU sociodemographic characteristic was significant.

### Multivariate results

Multivariate models were conducted to assess how much variability in area-level smoking was accounted for by all the area-level characteristics combined. Multivariate models predicting overall lifetime smoking (boys and girls combined)Multivariate models were conducted to assess how much variability in area-level smoking was accounted for by all the area-level characteristics combined. Multivariate models predicting overall lifetime smoking (boys and girls combined) and past 30-day smoking among girls each included 6 independent variables: 1) percentage of the population with less than a high school education, 2) percentage of residents living in an urban area, 3) median housing value, 4) median household income, 5) percentage of residents who were unemployed, and 6) percentage of residents with an income-to-poverty ratio less than 1.5. The model predicting mean lifetime prevalence of smoking was significant (F [6,53] = 4.18, *P* = .002), with the GPU sociodemographic characteristics accounting for 32% of the variability in mean lifetime smoking prevalence. The overall model predicting mean prevalence of past 30-day smoking among girls was also statistically significant (F [6,53] = 3.29, *P* = .008), with the GPU sociodemographic characteristics accounting for 27% of the variance. A multivariate model was not calculated for boys because none of the GPU sociodemographic characteristics was significantly associated with mean prevalence of past 30-day smoking in bivariate analyses (Table 2). In the multivariate models, none of the individual GPU-level sociodemographic characteristics was independently associated with mean smoking prevalence, after controlling for all other GPU sociodemographic characteristics. This is probably because of the high correlation among some of the predictor variables, which ranged from ±.05 to ±.93. The high correlations observed, however, do not affect the interpretation of the *r* ^2^ value (the amount of variability explained by the area-level sociodemographic characteristics).

## Discussion

This study was designed to further understand how macro-level factors are associated with variability in area-level smoking prevalence rates. Our results show that adolescent smoking rates vary greatly within states. To date, most tobacco surveillance systems assess smoking rates at the national or state levels (19). These results show that state-level estimates of adolescent smoking prevalence may mask large variation within states and that surveillance data need to include prevalence rates and trends in cities, counties, and communities within states. These data would be valuable in several ways. First, recent funding cuts for tobacco control programs emphasize the need to allocate resources to areas with the greatest need. Our results suggest that some communities may be in greater need of tobacco control programs than others. Local-level data are also critical for evaluation of tobacco prevention programs because the success of tobacco prevention programs is often measured by the changes in proportion of youth who smoke in the area targeted by the program (20).

We also found that communities characterized by low socioeconomic status, including lower overall education, lower median housing value, lower median household income, and higher unemployment, showed higher mean prevalence rates of youth smoking. GPUs characterized as more rural also had a higher prevalence rates of both lifetime and past 30-day smoking among youth. Combined, these social indicators explained a substantial proportion of the variation in smoking prevalence. Although others have reported on the association between social disparities and disease outcomes and premature mortality at the neighborhood level (21,22), rarely are these disparities reported for the prevalence of risk behaviors, such as smoking, and even more rarely are they reported for the adolescent population. We were able to find only 2 reports of area-level disparities in smoking prevalence for adults (4,5) and have found no such reports for adolescent smoking prevalence. Because disparities in smoking prevalence may lead to disparities in disease outcomes and mortality and because smoking is a behavior that largely begins in adolescence, attention to area-level variations in the prevalence of smoking, especially during adolescence, and their associations with area-level characteristics are likely to reduce later disparities in disease and death rates.

We also found that the relationship between GPU social and geographic characteristics and mean prevalence of past 30-day smoking differed for boys and girls. While only a weak relationship was found between any of the GPU sociodemographic characteristics and past 30-day smoking rates for boys, 6 of the 10 GPU characteristics were significantly related to rates of past 30-day smoking for girls. These results suggest that smoking rates for girls may be more sensitive to area-level characteristics than smoking rates for boys.

Our study has several limitations. First, although the number of areas included in our study was larger than in previous studies, a sample size of 60 is still somewhat limited. Second, we found a small amount of variability in some of the GPU-level characteristics, such as the percentage of residents who are white and who speak English, which may have limited our ability to detect relationships between these characteristics and youth smoking rates. The limited variability in some of the GPU-level characteristics might also limit the generalizability of these findings. Finally, our GPUs are variable in geographic size and definition. However, the GPUs are similar in terms of percentage of teenagers, and the GPUs are aggregates of block groups, which are similar to the census tracts recommended as an appropriate level of geography for measuring social disparities in health (23). Relationships at the GPU level, however, may not apply to other area-level units.

In conclusion, area-level characteristics may play an important role in adolescent smoking, particularly for girls. Recognition of these social disparities and environmental factors could have implications for future health and indicates the need for targeting smoking prevention programs to local-level needs.

## Figures and Tables

**Table 1:** Smoking Prevalence in 60 Geopolitical Units (GPUs) by GPU Characteristic, Minnesota Adolescent Community Cohort, October 2000-March 2001

GPU Characteristic	Mean (SD)	Range	Median	CV^a^, %
White, %	89.4 (14.0)	19.7-98.8	93.6	15.7
<High school degree, %	13.2 (6.0)	2.7-30.3	12.4	45.5
Married, %	54.2 (9.7)	25.4-68.9	57.7	17.9
Live in an urban area, %	65.5 (33.5)	0-98.8	75.8	51.1
Aged ≥18 y and speak English, %	92.3 (5.3)	72.5-97.5	93.7	5.7
Median housing value^b^, $	117.6 (43.8)	57.3-229.2	105.6	37.2
Live in renter-occupied housing, %	24.5 (12.2)	4.3-62.8	21.3	49.8
Median household income^b^, $	48.5 (14.7)	27.7-83.5	41.9	30.3
Aged ≥16 y and unemployed, %	4.5 (2.2)	1.7-14.2	3.8	48.9
Income-to-poverty ratio <1.5, %	15.7 (8.7)	4.0-47.4	14.3	55.4
**Community-level smoking, %**				
Ever smoked	33.1 (8.6)	13.3-53.3	NA	26.0
Past 30-day smoking	9.5 (3.9)	3.2-18.6	NA	41.1

Abbreviations: SD, standard deviation; CV, coefficient of variation; NA, not applicable.

a CV calculated as (SD/mean) x 100.

b Median housing and median household income values were divided by $1,000.

**Table 2:** Mean Percentage Prevalence of Lifetime and Past 30-Day Smoking by Median Split of Geopolitical Unit (n = 60) Characteristics, Minnesota Adolescent Community Cohort, October 2000-March 2001

GPU Characteristic	Ever Smoked		Past 30-Day Smoking			

	Mean % (SD)	*P* Value	Men, Mean % (SD)	*P* Value	Women, Mean % (SD)	*P* Value
**White **						
≤Median	32.5 (9.9)	.59	9.0 (5.6)	.58	9.3 (4.9)	.09
>Median	33.7 (7.2)		8.2 (5.4)		11.5 (5.0)	
**<High school degree **						
≤Median	28.4 (7.8)	<.001	7.8 (4.8)	.23	8.1 (4.5)	<.001
>Median	37.7 (6.8)		9.3 (6.0)		12.7 (4.4)	
**Married**						
≤Median	32.5 (9.6)	.60	8.3 (5.0)	.69	9.6 (4.9)	.25
>Median	33.7 (7.6)		8.9 (5.9)		11.1 (5.1)	
**Live in an urban area**						
≤Median	35.9 (6.7)	.01	8.9 (5.2)	.68	12.3 (4.5)	.003
>Median	30.3 (9.5)		8.3 (5.7)		8.5 (4.9)	
**Aged ≥18 y and speak English**						
≤Median	31.0 (9.3)	.06	7.7 (5.0)	.20	9.3 (5.1)	.10
>Median	35.2 (7.4)		9.5 (5.8)		11.5 (4.8)	
**Median housing value**						
≤Median	37.1 (7.6)	<.001	9.1 (6.0)	.49	12.3 (4.3)	.003
>Median	29.1 (7.8)		8.1 (4.9)		8.5 (5.0)	
**Live in renter-occupied housing**						
≤Median	33.7 (7.0)	.61	8.7 (5.5)	.90	11.3 (4.7)	.18
>Median	32.5 (10.1)		8.5 (5.5)		9.5 (5.2)	
**Median household income**						
≤Median	37.0 (7.6)	<.001	8.9 (6.1)	.66	12.4 (4.5)	.001
<Median	29.2 (7.8)		8.3 (4.8)		8.4 (4.8)	
**Aged ≥16 y and unemployed**						
≤Median	30.1 (7.3)	.006	7.7 (5.0)	.20	8.3 (4.6)	.001
<Median	36.1 (9.0)		9.5 (5.8)		12.5 (4.6)	
**Income-to-poverty ratio <1.5**						
≤Median	29.1 (7.8)	<.001	8.2 (4.8)	.62	8.2 (4.6)	<.001
>Median	37.0 (7.6)		8.9 (6.1)		12.6 (4.5)	

Abbreviation: SD, standard deviation.
